# The critical role of epigenetic mechanism in PM_2.5_-induced cardiovascular diseases

**DOI:** 10.1186/s41021-021-00219-w

**Published:** 2021-10-15

**Authors:** Qinglin Sun, Xiaoke Ren, Zhiwei Sun, Junchao Duan

**Affiliations:** 1 Department of Toxicology and Sanitary Chemistry, School of Public Health, Capital Medical University, 100069 Beijing, P.R. China; 2 Beijing Key Laboratory of Environmental Toxicology, Capital Medical University, 100069 Beijing, P.R. China; 3 School of Public Health, Capital Medical University, 100069 Beijing, P.R. China

**Keywords:** PM_2.5_, Cardiovascular disease, DNA methylation, m^6^A RNA methylation, Non-coding RNA, Histone modification, Chromosome remodeling

## Abstract

Cardiovascular disease (CVD) has become the leading cause of death worldwide, which seriously threatens human life and health. Epidemiological studies have confirmed the occurrence and development of CVD are closely related to air pollution. In particular, fine particulate matter (PM_2.5_) is recognized as an important environmental factor contributing to increased morbidity, mortality and hospitalization rates among adults and children. However, the underlying mechanism by which PM_2.5_ promotes CVD development remains unclear. With the development of epigenetics, recent studies have shown that PM_2.5_ exposure may induce or aggravate CVD through epigenetic changes. In order to better understand the potential mechanisms, this paper reviews the epigenetic changes of CVD caused by PM_2.5_. We summarized the epigenetic mechanisms of PM_2.5_ causing cardiovascular pathological damage and functional changes, mainly involving DNA methylation, non-coding RNA, histone modification and chromosome remodeling. It will provide important clues for exploring the biological mechanisms affecting cardiovascular health.

## Background

Fine particulate matter (PM_2.5_, the aerodynamic diameter less than 2.5 μm) in air pollution is a major public health problem worldwide. There is increasing evidence that air pollution affects public mortality [1]. According to the Global Burden of Disease (GBD) report, 7.5 % of global deaths (11.1 % in China) are attributable to air pollution [2, 3], and PM_2.5_ concentration increases by 10 µg/m³, the all-cause mortality rate will increase by 0.68 % [4]. Interestingly, recent studies have found long-term exposure to PM_2.5_ levels well below current World Health Organization standards, but the overall mortality rate of the population and the mortality rate of specific causes are still increasing [5]. There is a positive correlation between fine particulate matter and the negative health effects of humans such as respiratory diseases, cardiovascular diseases (CVD), and lung cancer. In recent years, the prevalence of CVD has continued to rise worldwide. In 2019, 6.2 million people in the 30 to 70 age group died from CVD [6]. The “Air Pollution and Cardiovascular Diseases” issued by the American Heart Association clearly pointed out that PM_2.5_ is one of the controllable risk factors leading to cardiovascular events [7]. Factors such as gender, age, and climate can affect the health effects of air pollution on the population, but epidemiological surveys show that CVD is the most closely related cause of death with air pollution [8]. Although the pollution of fine particulate matter has been actively controlled in China, it is still more serious compared with other areas [9].

In recent decades, investigators have conducted extensive studies on the relationship between air pollution and CVD, but the mechanism of PM_2.5_ causing cardiovascular damage is still not very clear. At present, a large number of clinical experiments and animal experiments have revealed relevant pathophysiological mechanisms, mainly including oxidative stress, systemic inflammation, vascular endothelial damage, mitochondrial damage, atherosclerosis, and changes in autonomic nerve function [10–12], among which oxidative stress may play an important role in the initiation and development of other mechanisms. Studies have shown that PM_2.5_ can induce and activate the production of reactive oxygen species and nitric oxide in cardiovascular endothelial cells [13, 14]. Its interaction triggers lipid peroxidation and changes the permeability and fluidity of cell membrane by oxidizing polyunsaturated fatty acids on the cell membrane. At the same time, the accumulation of oxidized lipids under the vascular endothelium and its secondary inflammation will promote the formation of plaques, causing atherosclerosis and impairing cardiovascular health.

Recently, a large number of evidences have suggested that epigenetics could be involved in the occurrence and development of CVD and play an important regulatory function in the cardiovascular system. Epigenetic changes are genetic changes in phenotype or gene expression when the DNA sequence has not changed, emphasizing the interaction between genes and the environment. Epigenetic modification imbalance is involved in the pathogenesis of cardiac hypertrophy and heart failure, regulating the transcriptional activity of transcription factors related to cardiac development [15, 16]. Studies have shown that epigenetic modifications may act as a bridge between oxidative stress and atherosclerosis [17]. External stimuli affect gene expression in endothelial cells, smooth muscle cells and macrophages. Subsequently, it may lead to epigenetic mutations, which eventually lead to the development of atherosclerosis [18–20].

**Fig. 1:**
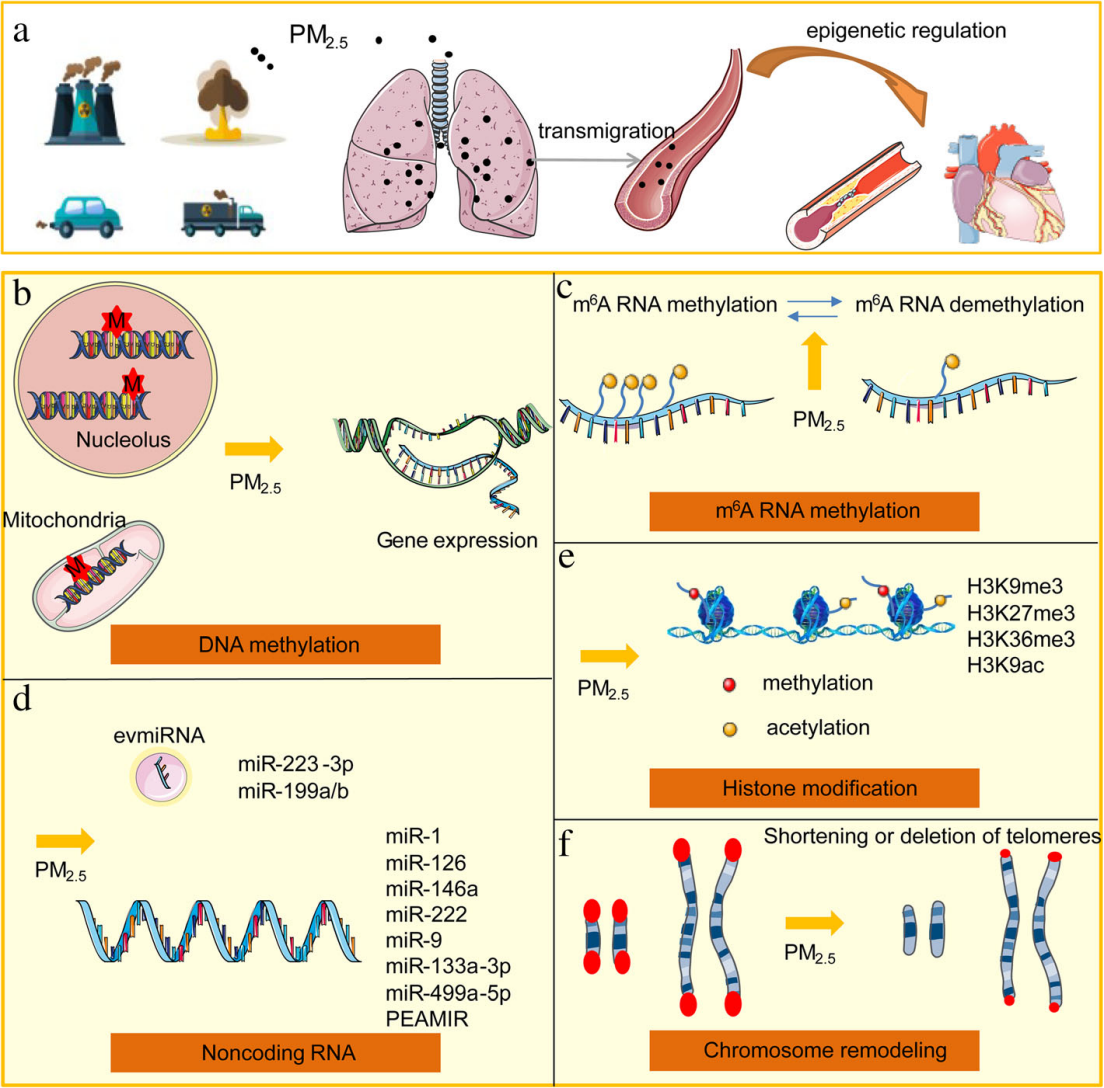
Epigenetic mechanisms involved in PM_2.5_-induced CVD. **a**) PM_2.5_ induced CVD through epigenetic mechanisms; Mainly involved five aspects: (**b**) DNA methylation; (**c**) m^6^A RNA methylation; (**d**) Non-coding RNA; (**e**) Histone modification; (**f**) Chromosome remodeling

In recent years, studies have found that epigenetic regulation is closely related to the cardiovascular hazards after PM_2.5_ exposure, such as histone acetylation modification, interferon γ methylation [21–23]. Exploring the epigenetic regularity of CVD caused by air pollution will help to understand the occurrence of diseases that interact with genes and the environment (Fig. 1a). The mechanism provides clues for research on the impact of air pollution on health. Gene regulation mediated by DNA methylation, m^6^A RNA methylation, non-coding RNA, histone modifications and chromosome remodeling are the most studied epigenetic mechanisms, which will be discussed in this review.

### DNA methylation

DNA methylation is an epigenetic regulatory mechanism that has been extensively studied, and it plays an important role in gene transcription. DNA methylation means that under the catalysis of DNA methyltransferase (DNMTs), S-adenosylmethionine provides a methyl group, which is added to the 5-carbon atom of cytosine-phosphate-guanine (CpG) dinucleotide by covalent bond [24]. The establishment and maintenance of DNA methylation in mammals involve three types of DNMTs, namely DNMT1, DNMT3A, and DNMT3B2 [25], which are involved in DNA methylation to regulate normal biological functions, such as embryonic development, cell differentiation, and gene transcription. Recent studies have found that DNA methylation in gene promoter regions and important regulatory sequences will not only block the expression of downstream related genes, but may also have an activating effect [26]. The dynamic balance between methylation and demethylation is an important condition for maintaining the normal homeostasis of the body’s epigenetics. When this dynamic balance is broken, DNA is abnormally methylated, and some genes will be overexpressed or inhibited, leading to disease. DNA methylation is involved in the process and outcome of CVD. A study based on five population cohorts shows that gene methylation has a potential causal relationship with CVD [27]. Recent evidence links DNA methylation to cardiovascular hazards after PM_2.5_ exposure [28, 29], revealing that changes in DNA methylation may be a potential tool for CVD diagnosis.

More than 90 % of methylated CpG sites in the human genome occur in repetitive sequences, especially Alu sequences and long interspersed nuclear elements (LINE-1). The methylation levels of LINE-1 and Alu can be used as surrogate markers of overall genomic DNA methylation [30]. In normal differentiated cells, the LINE-1 promoter is hypermethylated and does not transpose, while it is activated in the early stages of germ cell development and embryonic development [31, 32]. Studies have found that LINE-1 hypomethylation is associated with increased morbidity and mortality of CVD [33]. In a randomized double-blind crossover trial of 35 healthy college students from Shanghai, China, researchers observed that exposure to PM_2.5_ in the air can induce a rapid decrease in DNA methylation in the repetitive elements LINE-1, Alu, and certain specific genes. An interquartile range increase (64 µg/m^3^) in PM_2.5_ was significantly associated with reduction of methylation in LINE-1 (1.44 %), one pro-inflammatory gene (CD40LG encoding soluble CD40 ligand, 9.13 %), two pro-coagulant genes (F3 encoding tissue factor, 15.20 %; SERPINE1 encoding plasminogen activator inhibitor-1, 3.69 %), and two pro-vasoconstriction genes (ACE encoding angiotensin-converting enzyme, 4.64 %; EDN1 encoding endothelin-1, 9.74 %). It was discovered for the first time that gene-specific hypomethylation plays an important role in the process of PM_2.5_ causing changes in biomarkers such as cardiovascular system inflammation, coagulation and vasoconstriction [28]. In the standard aging cohort, Andrea et al. used pyrosequencing to perform methylation sequencing on the peripheral blood samples of 704 elderly men. Assessing the impact of particulate matter exposure in different phases on methylation, it is found that methylation of repeated elements, such as LINE-1, was significantly reduced after short-term exposure to PM_2.5_. Furthermore, the researchers also found a positive correlation between LINE-1 hypomethylation and inflammation effects [34, 35]. In addition, a cross-sectional study conducted in Italy by Martina et al. showed that exposure to PM was negatively correlated with LINE-1 methylation in healthy women. In particular, they reported that the methylation level of LINE-1 decreased significantly with the increase of age [36].

A series of studies have provided valuable data. In an epidemiological study just released, researchers demonstrated that acute exposure to PM_2.5_ affects the methylation of pro-inflammatory genes and changes the function of cardiac parasympathetic regulation [21]. Interestingly, some studies have evaluated the relationship between DNA methylation and prenatal exposure. In a longitudinal cohort study, researchers have observed that prenatal exposure to PM_10_ and PM_2.5_ is related to changes in the methylation of a few gene promoters in the blood of newborns [37]. Some of these genes are also related to cardiovascular health outcomes in later childhood. Similarly, Russell and his colleagues found that C57BL/6J mice exposed to particulate matter during pregnancy had increased methylation of the HSD11B2 gene promoter and abnormal blood pressure in their offspring [38]. An epidemiological study on clock genes showed that exposure to high concentrations of PM_2.5_ can significantly change the methylation patterns of genes that regulate the circadian rhythm pathway, thereby increasing the risk of stroke events [39]. By contrast, a longitudinal panel study showed that a significant negative correlation between PM_2.5_ exposure and ACE methylation. Increased ACE protein accounted for 3.90~13.44 % of the elevated blood pressure by PM_2.5_. Therefore, it is speculated that hypomethylation of ACE gene may be one of the main epigenetic mechanisms of PM_2.5_ increasing blood pressure [29]. In addition, studies have analyzed the effects of PM_2.5_ on cardiomyocytes using methylation chips, and found that genes with methylation changes are significantly concentrated in pathways in regulation of apoptotic process, cell death and metabolic pathways [40]. This provides additional insights for studying the underlying mechanism of PM_2.5_-related heart disease.

In addition to nuclear DNA, PM_2.5_ also affects mitochondrial DNA (mtDNA) methylation. In a study of two groups of people with low and high exposure to environmental particles, the results suggest that locus-specific mtDNA methylation is correlated to PM exposures and mtDNA damage. Specifically, steel workers with high exposure to metal-rich PM exhibited higher levels of three specific mtDNA loci, i.e., the transfer RNA phenylalanine (MT-TF), 12 S ribosomal RNA (MT-RNR1) gene and “D-loop” control region [41]. Hyang-Min et al. found that mtDNA methylation in the blood of people exposed to PM_2.5_ is negatively correlated with PM_2.5_ concentration, and also found that the heart rate variability of subjects with higher mtDNA methylation levels is more likely to be affected by PM_2.5_ [42]. As we all know, heart rate variability is a very valuable indicator for predicting sudden cardiac death and arrhythmia events, and can be used to determine the condition and prevention of CVD [43]. In recent years, some studies have begun to pay attention to the relationship between PM and heart rate variability to speculate on important risk factors for CVD [44, 45].

In summary, the role of DNA methylation in the occurrence and development of CVD caused by air pollution exposure has received increasing attention. PM_2.5_ may lead to transcriptional activation of some CVD-related genes by promoting promoter demethylation (Fig. 1b). However, the relationship between PM_2.5_ exposure and DNA methylation has not reached a consistent conclusion. It is still necessary to further search for DNA methylation sites related to air pollution that leads to CVD as intervention targets to reduce the harm of PM_2.5_ exposure to the human body.

### m^6^A RNA methylation

N6-methyladenosine (m^6^A) methylation is one of the most common epigenetic modifications of eukaryotic RNA [46]. It is a methylation modification formed by the 6th nitrogen atom of adenine through the catalysis of methylase, a newly discovered post-transcriptional gene expression regulation. The dynamic and reversible modification of m^6^A is accomplished by m^6^A methyltransferase, m^6^A demethylase, and m^6^A binding protein, which are separately described as ‘writers’, ‘erasers’ and ‘readers’ [47]. Messenger RNA (mRNA) is methylated under the catalysis of the ‘writer’, and this process can be reversed under the catalysis of the ‘eraser’. Moreover, the methylated mRNA can be recognized by the ‘reader’. The ‘writer’ methyltransferase is a complex composed of methyltransferase like 3 (METTL3), METTL14 and Wilms’ tumor 1-associating protein (WTAP) [48]. The main components of m^6^A demethylase include fat mass and obesity associated (FTO) genes and α-ketoglutarate-dependent dioxygenase alkB homolog 5 (ALKBH5) [49, 50]. The components of m^6^A binding protein include members of the YTH domain protein family and heterogeneous nuclear ribonucleoprotein (HNRNP). Among them, the former includes two subtypes of YTH domain family protein (YTHDF) and YTH domain containing (YTHDC). The binding proteins of YTHDF subtypes are YTHDF1, YTHDF2 and YTHDF3, and the binding proteins of YTHDC subtypes are YTHDC1 and YTHDC2 [51]. Compared with DNA methylation, m^6^A can more directly regulate gene transcription and translation, realizing the rapid response to changes in the external environment. The researchers analyzed the expression of genes involved in the regulation of RNA methylation in people exposed to high or low concentrations of PM_2.5_. Expression of the writers METTL3 and WTAP were 1.27- and 2.11-fold higher in the high-PM_2.5_ group in comparison to the control group. Similarly, expression of the erasers FTO and ALKBH5 were 1.31- and 1.29-fold higher in the high-PM_2.5_ group in comparison to the control group, while the reader gene HNRNP was 1.52-fold higher in the high-PM_2.5_ group [52]. And this is the first study of the effect of environmental particulate matter on m^6^A RNA methylation. However, a cohort study examined the association of smoking and particulate matter with peripheral blood RNA m^6^A. Results showed that ever smoking was associated with a relative 10.7 % decrease in global m^6^A in men in comparison to nonsmokers. Surprisingly, global m^6^A was not associated with acute exposure to PM_2.5_ [53]. Even so, future studies should not ignore the association between m^6^A and PM_2.5_ exposure in vivo, and should also explore the impact of differences in the duration of PM_2.5_ exposure. In recent years, many studies have found that m^6^A RNA methylation plays an important role in the pathogenesis of heart failure, cardiac hypertrophy, aneurysm, pulmonary hypertension and vascular calcification [54–56]. Exposure to airborne particulate matter may cause dynamic changes in m^6^A methylation and demethylation, affecting gene transcription (Fig. 1c). Therefore, m^6^A is expected to serve as a bridge between PM_2.5_ exposure and CVDs. This provides a new perspective for the study of the mechanism of PM_2.5_ causing cardiovascular toxicity.

### Noncoding RNA

Non-coding RNAs (ncRNAs) have important functional significance in human life, health and disease diagnosis and treatment. There are many types of ncRNAs classified by function. Among them, functional ncRNAs that are not translated into protein mainly include MicroRNA (miRNA), Long ncRNA (lncRNA) and Circular RNA (circRNA). ncRNAs participate in a variety of CVD and other diseases by controlling DNA methylation, influencing histone modification, and regulating mRNA transcription, stability and translation [57, 58]. Moreover, the abnormal expression of ncRNA can cause cell dysfunction, which can be used to reveal the relationship between environmental particulate matter and adverse cardiovascular events [59].

miRNAs play an important regulatory role in the biological processes of cell proliferation, apoptosis and differentiation [60]. Recent studies have found that PM_2.5_ can affect the expression of microribonucleic acid and produce potential biological effects [61]. In a study of elderly men exposed to environmental particulate matter for different periods of time, Serena and his colleagues found that PM_2.5_ was significantly negatively correlated with miR-1, miR-126, miR-146a, miR-222, and miR-9. It is worth noting that this effect is most obvious on the 7th day, suggesting that short-term exposure to air particles can cause rapid changes in miRNA [62]. Previous studies have shown that miR-1, miR-126, and miR-222 regulate important biological pathways in the cardiovascular system and participate in the occurrence and development of atherosclerosis and other CVD [63, 64]. Julian et al. tested the levels of miRNAs in the plasma of people exposed to airborne particulates to find the link between PM_2.5_ exposure and miRNA. Compared with people exposed to low levels of PM_2.5_, they observed decreased miR-133a-3p levels in 95 % of the subjects who exposed to high levels of PM_2.5_, in 85 % decreased miR-193b-3p levels, in 80 % increased miR-1224-5p levels, in 85 % decreased miR-433-3p levels, in 80 % decreased miR-145-5p levels, in 65 % decreased miR-27a-5p levels, in 60 % decreased miR-580-3p levels, in 55 % increased miR-3127-5p levels and in 75 % decreased miR-6716-3p levels. Among the rest, low expression of miR-133a-3p and miR-145-5p is associated with CVDs [65]. This down-regulation may cause serious consequences such as cardiac hypertrophy, severe fibrosis, and heart failure [66, 67]. In addition, a study of 55 healthy students exposed to indoor PM_2.5_ showed that PM_2.5_ may promote the pathological development of CVD by inducing systemic inflammation, coagulation, vasoconstriction, or the expression of cytokines in endothelial dysfunction. Among them, miRNAs play an important role in these regulatory processes [11].

Extracellular vesicles are rich in miRNAs, can be detected in readily available blood and body fluids, and are non-invasive [68], which is of great significance for the in-depth understanding of the pathogenesis of PM_2.5_-induced CVD. A study collected the sera of 22 veterans and confirmed that long-term exposure to PM_2.5_ upregulated miR-223-3p and miR-199a/b in extracellular vesicle miRNAs (evmiRNAs). It also suggests that they are involved in the process of oxidative stress, inflammation, atherosclerosis, and blood pressure [22, 69]. Recently, studies have shown that short-term exposure to particulate matter also increases the release of evmiRNAs [70], suggesting that evmiRNAs may become a marker of particulate matter susceptibility.

Previous studies have shown that lncRNA has an irreplaceable role in the normal physiological functions of the cardiovascular system [71]. Recently, more and more studies have provided evidence that lncRNA is involved in the pathogenesis of PM_2.5_-induced CVD. Pei et al. found that PM_2.5_ exposure can reduce the expression of LncRNA PEAMIR through in vivo and in vitro experiments [72]. It is worth noting that PEAMIR can effectively bind to key miRNAs in myocardial ischemia/reperfusion injury and exert an inhibitory effect. The down-regulation of PEAMIR increases the risk of myocardial ischemia/reperfusion injury. Other studies have shown that some lncRNAs play a regulatory role in PM_2.5_-mediated endothelial cell inflammation, such as NONHSAT247851.1. This finding helps to understand the occurrence and development of inflammatory vascular diseases [12]. Up to now, there are no reports about the role of circRNA in PM_2.5_-induced CVD. In summary, PM_2.5_ can up-regulate or down-regulate the expression of ncRNA in the body, and activate downstream related signal molecules, thus causing damage to the cardiovascular system (Fig. 1d).

### Histone modification

Histones are a key factor in keeping chromatin in an inhibited or active conformation. Histone subunits are octamers composed of two molecules each of H2A, H2B, H3, and H4 [73], which can be tightly combined with acidic DNA and are the main components of the basic unit of eukaryotic chromatin. Histones can undergo modifications such as methylation, acetylation, phosphorylation, ubiquitination, etc., and they can aggregate in different modes to regulate chromatin structure. Unlike DNA methylation, the effects of histone modifications on gene expression may vary due to specific chemical modifications [74]. In recent years, more and more studies have confirmed that environmental factors are related to histone modifications. Abnormal histone modifications can in turn lead to many diseases, including CVD.

Studies have proved that histone methylation has a complicated relationship in maintaining the cell epigenome, and histone methylation modification is mediated by histone methyltransferases (HMTs) [75]. Zheng et al. observed the impact of traffic-derived particulate matter exposure on the health effects of truck drivers and found that PM is related to several types of histone H3 modifications in blood leukocytes. Namely H3 lysine 9 trimethylation (H3K9me3), H3 lysine 27 trimethylation (H3K27me3), H3 lysine 36 trimethylation (H3K36me3) and H3 lysine 9 acetylation (H3K9ac). The researchers also showed diminishing utility of these histone biomarkers of blood pressure throughout the day, particularly among individuals with occupational exposures [76]. This is the first epidemiological study to study the relationship between histone modification and blood pressure. Recently, histone acetylation and deacetylation are also considered to be one of the most important regulatory mechanisms that mediate cardiovascular development and myocardial damage. Histone deacetylation is involved in the regulation of gene transcription under stress or pathological conditions [77]. In a study of workers in an Italian steel factory, Cantone and his colleagues observed that PM in the air is directly proportional to the extracellular plasma histone modification H3K9ac, and showed that histone modification mediates PM_2.5_ to cause blood coagulation [78]. In addition, histone acetylation also plays an important role in PM_2.5_ exposure-induced cardiac hypertrophy events. Studies have found that in the hearts of PM_2.5_-exposed mice, the level of acetylated H3K9 protein increased significantly, leading to the up-regulation of hypertrophic transcription factors [23, 79]. These findings suggest that the imbalance between histone methylation and demethylation as well as acetylation and deacetylation increase the possibility of cardiac dysplasia and diseases related to the cardiovascular system under PM_2.5_ air pollution conditions (Fig. 1e).

### Chromosome remodeling

Chromosome, as the genetic information of eukaryotic cells, is constantly damaged by various harmful factors in vivo and in vitro. People exposed to high levels of PM_2.5_ are at higher risk of developing certain diseases and living shorter lives. These negative effects may be caused by abnormal macromolecular changes caused by environmental pollutants, such as chromosomal aberrations [80]. Based on the analysis of baseline blood samples from 933 men ≥65 years of age from the prospective Cardiovascular Health Study, researchers have found that PM_10_ may increase white blood cell Y chromosomal mosaic deletions, a marker of genomic instability [81]. Studies have found a link between shorter telomere length and air pollution [82]. A longitudinal study found that pollution exposure is related to the length of chromosomal telomeres in children. When exposed to airborne particles, the telomeres in the cell will become longer and then shorter. In the later years of this group, the risk of chronic diseases will increase [83]. The accelerated erosion of telomere length leads to a decrease in the ability of cells to replicate in the heart or other tissues in the cardiovascular system, which may directly lead to the progression of CVD [84]. What’s more, the study found a stronger correlation between PM_2.5_ and shortened telomere length in white blood cells in girls than in boys during PM_2.5_ exposure [85]. These data suggest that PM_2.5_ may cause long-term changes such as chromosomal morphological abnormalities and shortening of telomeres on chromosomes (Fig. 1f), which have profound effects on cardiovascular morbidity and mortality in the population.

## Conclusions

The epigenetic study in PM_2.5_-induced CVD is still preliminary, and it is urgent to verify the causal role of many epigenetic changes in particulate matter-related CVD. With the discovery of more types of epigenetic regulation in the human genome, the field of epigenetic regulation is still expanding. Many studies focused on CVD are encouraged to consider the potential role of epigenetics, which involves DNA methylation, histone modification and miRNA expression. In-depth discussion of the role of epigenetic regulation in CVD is the direction of future research, providing a theoretical basis for the treatment of CVD caused by environmental pollution.

## Data Availability

Not applicable.

## Abbreviations

CVD: cardiovascular disease
PM_2.5_: fine particulate matter
GBD: Global Burden of Disease
DNMTs: DNA methyltransferase
CpG: cytosine-phosphate-guanine
LINE-1: long interspersed nuclear elements
ACE: angiotensin-converting enzyme
mtDNA: mitochondrial DNA
MT-TF: transfer RNA phenylalanine
MT-RNR1: 12 S ribosomal RNA
m^6^A: N6-methyladenosine
METTL3: methyltransferase like 3
WTAP: Wilms’ tumor 1-associating protein
FTO: fat mass and obesity associated
ALKBH5: α-ketoglutarate dependent dioxygenase alkB homolog 5
HNRNP: heterogeneous nuclear ribonucleoprotein
YTHDF: YTH domain family protein
YTHDC: YTH domain containing
ncRNAs: non-coding RNAs
miRNA: MicroRNA
lncRNA: long ncRNA
circRNA: circular RNA
evmiRNAs: extracellular vesicle miRNAs
HMTs: histone methyltransferases
H3K9me3: H3 lysine 9 trimethylation
H3K27me3: H3 lysine 27 trimethylation
H3K36me3: H3 lysine 36 trimethylation
H3K9ac: H3 lysine 9 acetylation
